# Developing current procedural terminology codes that describe the work performed by machines

**DOI:** 10.1038/s41746-022-00723-5

**Published:** 2022-12-03

**Authors:** Richard A. Frank, Robert Jarrin, Jordan Pritzker, Michael D. Abramoff, Michael X. Repka, Pat D. Baird, S. Marlene Grenon, Megan Ruth Mahoney, John E. Mattison, Ezequiel Silva

**Affiliations:** 1 American Medical Association’s Digital Medicine Payment Advisory Group (DMPAG) AI Workgroup, American Medical Association, Chicago, IL USA; 2 Frank Healthcare Advisors, LLC, Gainesville, FL USA; 3 The Omega Concern, LLC, Washington, DC USA; 4 CVS/Aetna, Inc., Hartford, CT USA; 5 University of Iowa, Iowa City, IA USA; 6 Digital Diagnostics, Inc, Coralville, IA USA; 7 Johns Hopkins University School of Medicine, Baltimore, MD USA; 8 Philips, Cambridge, MA USA; 9 University of California San Francisco, Innovation Ventures, San Francisco, CA USA; 10 University of California San Francisco, San Francisco, CA USA; 11 Arsenal Capital Partners, New York, NY USA; 12 South Texas Radiology Group, San Antonio, TX USA; 13 UT Health San Antonio, Long School of Medicine, Department of Radiology, San Antonio, TX USA

**Keywords:** Health services, Health policy

## Abstract

The “Taxonomy of Artificial Intelligence for Medical Services and Procedures” became part of the Current Procedural Terminology (CPT®) code set effective January 1, 2022. It provides a framework for discrete and differentiable CPT codes which; are consistent with the features of the devices’ output, characterize interaction between the device and the physician or other qualified health care professional, and foster appropriate payment. Descriptors include “Assistive”, “Augmentative”, and “Autonomous”. As software increasingly augments the provision of medical services the taxonomy will foster consistent language in coding enabling patient, provider, and payer access to the benefits of innovation.

## Introduction

Artificial Intelligence (AI) in health care had been discussed frequently within the American Medical Association’s House of Delegates when in 2019, this group accepted a report “Augmented Intelligence in Health Care”^1,2^. One of the ways that the American Medical Association (AMA) responded to this report is that the AMA’s Digital Medicine Payment Advisory Group (DMPAG) formed a workgroup with specific expertise in AI. The workgroup developed the Taxonomy of Artificial Intelligence for Medical Services and Procedures and made application to the Current Procedural Terminology (CPT®) Editorial Panel for this taxonomy to be included as an appendix within the CPT® code set^3^. The Editorial Panel accepted this application during their September 2021 meeting, and Appendix S was made effective January 1, 2022.

**Fig. 1:**
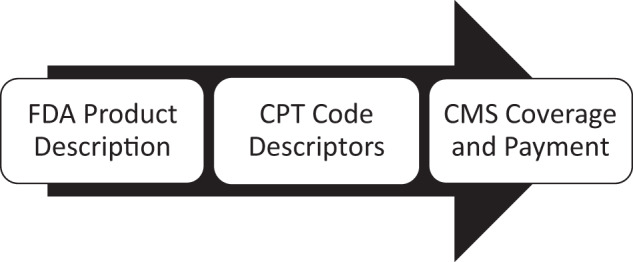
The continuum of AI CPT code descriptors. Appendix S is intended to enable discrete and differentiable code descriptors by accurately characterizing the “work done by machines” relative to the work of the physician or other qualified health care professional. Therefore, these same descriptors may be useful also in regulatory labeling of novel products like “Software as a Medical Device” (SaMD) and in valuation, coverage and payment policy for the associated novel services or procedures, like “Software as a Service” (SaaS).

CPT® is the most widely accepted medical nomenclature used across the country, offering physicians and health care professionals a uniform language for coding professional services and procedures. Public and private insurers rely on CPT codes to track and pay for medical services^4^. CPT codes are designed to describe the work performed by the physician. When the service is provided in a nonfacility setting (e.g., physician office), CPT codes also account for the practice expense^5^, both fixed and specific to the individual patient for an individual service. The factors considered in determining the value of physician work are the time it takes to perform the service, the necessary technical skill and physical effort, the required mental effort and clinical judgment, and stress due to the potential risk to the patient^6^. Appendix S provides language to describe the “work done by machines” in relation to the work of the physician. Although this concept is specific to CPT coding, the descriptors in Appendix S can help standardize terminology across healthcare; differentiation of the components of software-based medical service will facilitate labeling, valuation, coverage, and payment^7^ (Fig. 1). This article provides context and background on the taxonomy so that innovators, payors, regulators, medical professionals, and patient organizations can better understand it as they develop CPT code change applications (CCAs) for novel AI products and services.

The workgroup engaged in an heuristics exercise to identify AI products and services currently available or in development and compared them to a limited number of existing AI-related CPT codes such as multianalyte assays with algorithmic analyses and computer-aided detection imaging. The workgroup noted the large number of FDA-cleared devices not yet represented by a CPT code indicated a clear and present need for applicable descriptors to be used in CCAs^8^ The FDA has to date reviewed and authorized 343 “Software as a Medical Device (SaMD)”^9^ products that companies legally market via 510(k)clearance, de novo process, or pre-market approval. Developers and innovators have clinically beneficial products to bring to market and finding their place within CPT is a logical next step to ensure patients have access to the benefits of those software-based services.

The workgroup also considered a product for autonomous point of care diabetic retinal exam which had been cleared by FDA through their de novo pathway. For lack of any precedent in the code set, CPT code 92229 had been created using the word “automated” instead of “autonomous”. At their February 2022 meeting, shortly after Appendix S was made effective, the CPT Editorial Panel considered and adopted a CCA for revision of code 92229 by removing the term “automated” and replacing it with “autonomous”.

## Unique features of AI

The workgroup concluded that clinical integration and payment would require terminology and categorization for AI CPT code descriptor. With all this in mind the workgroup determined that, to describe the “work done by machines”, CPT codes for AI should be consistent with the technological features of the device or its output, characterize the interaction between the device and the physician or other qualified health care professional (QHP), be discrete and differentiable from other codes and descriptors, foster correct valuation, coverage, and appropriate payment, and support a reasonable range of business models and sites of service.

The taxonomy is applicable to both Category I and Category III CPT codes. Category I CPT codes provide a uniform language for coding medical services and procedures and Category III CPT codes are a set of temporary codes that allow data collection for emerging technologies, services, procedures, and service paradigms. As the “work done by machines” increasingly contributes to the provision and intensity of medical services, the taxonomy is meant to facilitate proper and accurate CPT coding, so the integration of AI into tracking, coverage, and payment systems evolves in a consistent manner. This will help facilitate patient access to the technology and accurate payment to physicians.

In some cases, rather than reducing physician input, the complexity of the output from AI may counterintuitively increase physician work, which currently may not be transparent in coding, coverage, and payment. The taxonomy is a framework intended to engender descriptors which are discrete and differentiable. Ideally, the resultant coding will do more than simply characterize the “work done by the machine” along the spectrum of “assistive,” “augmentative,” and “autonomous,” but will also delineate the machine’s part in the medical service and impact on the subsequent human work in such a way that the coding, coverage, and payment for the services will fairly reflect the contribution of each as shown in Table 1.

**Table 1:** The relationship between components of the service and categorization.

Service components	AI Category: Assistive	AI Category: Augmentative	AI Category: Autonomous
Primary Objective	Detects clinically relevant data	Analyzes and/or quantifies data in a clinically meaningful way	Interprets data and independently generates clinically meaningful conclusions
Provides independent diagnosis and/or management decision	No	No	Yes
Analyzes data	No	Yes	Yes
Requires physician or other QHP interpretation and report	Yes	Yes	No
Examples in CPT code set	Computer-aided detection (CAD) imaging (77048, 77049, 77065–77067, 0042 T, 0174 T, 0175 T)	Non-invasive estimate of coronary fractional flow reserve derived from coronary CT (7X005, effective Jan 2024, https://www.ama-assn.org/system/files/september-2022-cpt-summary-panel-actions.pdf)	Retinal imaging (92229)

## Current context

The AI Taxonomy, published on the AMA website and made effective from January 2022, provides guidance for choices of language for descriptors for AI-related CCAs^3^. It is intended that all AI-related CCA’s would be drafted and assessed with knowledge of the AI taxonomy henceforth, and descriptors used consistently across like codes.

When applicants submit a CCA for an AI-related code, the code change requestor should include technical details necessary to support the CPT Editorial Panel’s conclusion as to where the technology fits in the CPT-defined hierarchy of “assistive”, “augmentative”, and “autonomous”. Developers should consult the AI Taxonomy when developing descriptors for other highly sophisticated software. For example, Magnetic Resonance Spectroscopy is a highly sophisticated software, not the result of machine learning as is typical of products like the IDx-DR diabetic retinopathy device discussed *supra*, but instead is an “expert system” which renders data understandable in terms of intermediary metabolism associated with pathophysiology^10^. Such analysis of data is quite different, and adds more value, than simple image reconstruction. These types of “expert systems” may meet the criteria within the hierarchy of the taxonomy. As another example, deriving an estimate of Fractional Flow Reserve from coronary CT is considered augmentative because the quantitative output is clinically meaningful, ie understood by physicians to be directly applicable in optimizing the patient’s care pathway.

## Why no definition of the term “AI”?

In health care, there is no single product, procedure, nor service for which the term “AI” is sufficient or necessary to describe its intended clinical use or utility; and therefore, the specific term “AI” is not defined in the AI Taxonomy nor elsewhere in the CPT code set. The term “AI” is not intended to encompass nor constrain the full scope of innovations performing “work done by machines”, nor are these anthropomorphic terms intended to convey that the physician is being “replaced”^11^ Classification of AI medical services and procedures as assistive, augmentative, and autonomous is based on the clinical procedure or service provided to the patient, and the work performed by the machine on behalf of the physician or other QHP.

## Granularity for each of assistive, augmentative, and autonomous

This AI taxonomy is guidance for classifying various applications of AI (expert systems, machine learning, software-based services, etc.) medical services and procedures into one of three categories: assistive, augmentative, and autonomous. The workgroup selected these terms to characterize the work done by the machine relative to the work performed by the physician or other QHP. These words should become intuitive to stakeholders describing healthcare AI services in the CPT context.

Assistive services or procedures detect clinically relevant data; analysis and interpretation remain to be done and the physician work is not reduced. The data may be from other acquisition devices and transmitted via a third device. The service or procedure performed by the device “assists” the physician by bringing to their attention something which may or may not impact the diagnosis or management decision but is clinically relevant and might otherwise be missed. The physician does the remainder of the work—analyzes, determines the clinical importance of, and reports their interpretation of the data.

Augmentative services or procedures provide clinically meaningful analyses and/or quantification of data, without interpretation or reporting. The data may be acquired, transmitted, and detected by other devices. The physician may perform additional analyses but interprets the output from the analysis and reports conclusions based on their interpretation of the data. “Clinically meaningful” is in the judgement of a prudent physician that the output of the device will be directly applicable in optimizing their patient’s care pathway.

Autonomous services or procedures include interpretation and clinical conclusions which are independently reported and may lead directly to a recommendation and action, depending on the level of autonomy. A device performing procedures or services autonomously may or may not have acquired, transmitted, or detected the data.

The work performed by the machine for the physician or other QHP is autonomous when the machine automatically interprets data and independently generates clinically meaningful conclusions without concurrent physician or other QHP involvement. An autonomous medical service includes interrogating and analyzing data. The clinically meaningful conclusion may be a characterization of data (e.g., likelihood of pathophysiology) used to establish a diagnosis or to implement a therapeutic intervention. As illustrated in Table 2, there are three levels of autonomous AI medical services and procedures with varying physician or other QHP involvement:

1. The device interprets data, draws conclusions, and offers diagnosis and/or management options. These options are contestable and require action on the part of a physician or other QHP to implement.
2. The device interprets data, draws conclusions, and initiates diagnosis and/or management options with alert/opportunity for override, may require physician or other qualified QHP action to implement.
3. The device interprets data, draws conclusions, and initiates diagnosis and/or management options without requiring action by physician or other QHP to implement. The physician or QHP would have to take initiative to intervene or contest the action taken by the device.

**Table 2:** Levels of autonomy.

Level of autonomy	I	II	III
Interprets	+	+	+
Recommends		+	+
Acts			+

## Why is “automated” not in the taxonomy?

As discussed previously, the CPT Panel members used the term “automated” in 92229 because there was no precedent for autonomous as a descriptor; however, the term “automated” does not appropriately capture the “work done by machines” in AI-related services like the retinal exam. The term automated connotes a workflow efficiency obtained by utilizing a machine. Moreover, if a task or analysis is conducted automatically, then it may not be separable, there may have been no clinical judgement applied to the procedure or service for that patient, and, therefore, the clinical necessity may be unclear. For example, 3D volumetrics may be performed automatically on all chest CT images loaded to a PACS system, whereas the clinical utility of 3D volumetrics is only established for a subset of the patients undergoing that image acquisition.

## Future application

As technology develops and evolves, the application of guidance provided in the AI taxonomy should help to maintain consistency across CPT codes that describe the work performed by machines. Further, it may be necessary to modify this taxonomy framework. Consistency in the depiction of the interaction between physician and machine will be important in valuation even as innovation in this relationship between human and machine is likely to evolve.

Implementation of this taxonomy within the CPT code set will require intentionality by the CPT Editorial Panel, so the precedents are used consistently among similar healthcare AI. The AMA will continue to gather technical expertise to keep pace with technologies expected to improve patient and population outcomes, expand access to care, enhance patient experience, and promote physician satisfaction.

### Reporting summary

Further information on research design is available in the Nature Research Reporting Summary linked to this article.

## Supplementary information

Reporting Summary

## Data Availability

This perspective is based on the authors’ experience in developing CPT Appendix S: Artificial Intelligence Taxonomy for Medical Services and Procedures. Typically, recollections are not appropriate for a data repository. Questions may be directed to the corresponding author.
